# Anti-osteoporotic effect of sitagliptin in an osteoporosis model of ovariectomized rats: role of RUNX2 and RANKL/OPG ratio

**DOI:** 10.1007/s00210-025-04145-4

**Published:** 2025-05-21

**Authors:** Salma Ahmed El-Marasy, Rehab F. Abdel-Rahman, Reham M. Abd-Elsalam, Hanan A. Ogaly, Rasha M. Allam

**Affiliations:** 1 Department of Pharmacology, Medical Research and Clinical Studies Institute, National Research Centre, Giza, Egypt; 2 Department of Pathology, Faculty of Veterinary Medicine, Cairo University, Giza, Egypt; 3 Department of Biochemistry and Chemistry of Nutrition, Faculty of Veterinary Medicine, Cairo University, Giza, Egypt

**Keywords:** Sitagliptin, Ovariectomy, RANKL/OPG, RUNX2, TRAP, Rats

## Abstract

This study examines the potential anti-osteoporotic effect of sitagliptin in osteoporosis instigated by ovariectomy (OVX) in rats. Rats were assigned into 4 groups: Sham-operated, OVX group, and OVX rats orally treated with sitagliptin (10, 20 mg/kg), respectively, after 8 weeks of OVX for 4 weeks. Biochemical, real-time polymerase chain reaction, histopathological, and immunohistochemical analyses of bone resorption and formation were conducted. Sitagliptin ameliorated bone mineral density (BMD), restored calcium and phosphorus levels in OVX rats, elevated catalase and decreased malondialdehyde, reduced receptor activator of NF-κB ligand (RANKL), elevated osteoprotegerin (OPG), and reduced tartrate-resistant acid phosphatase (TRAP) femur contents. Sitagliptin mitigated variations in mRNA expressions of RUNX2 and protein kinase B (AKT) in femur tissue. Moreover, sitagliptin reduced caspase-3 protein expression and improved bone histomorphology and mechanical properties. Sitagliptin’s anti-oxidant activity mediated its anti-osteoporotic effect in OVX rats via modulation of RUNX2, downregulation of RANKL/OPG, AKT pathways, apoptosis, and histomorphometry alterations revealing attenuation of osteoclastogenesis and promotion of osteoblast formation.

## Introduction

Osteoporosis is a systemic metabolic bone disease characterized by progressive loss of bone mass, deterioration of bone microstructure, and increased risk of fracture (Ekeuku et al. 2022). It is also a rising global public health concern with over 9 million osteoporotic fractures occurring annually (Lu et al. 2022). Postmenopausal osteoporosis is the most common type of osteoporosis, due to the sudden decline of estrogen levels in women, which enhances the differentiation and survival of osteoblasts and inhibits the same processes in osteoclasts (Xu et al. 2022).

Bone remodeling is a pivotal process in the etiology of osteoporosis. Bone remodeling is held by osteoblast controlling bone formation and osteoclast regulating bone resorption (Han et al. 2019). Bone remodeling is controlled by the receptor activator of NF-κB (RANK)/RANK ligand, (RANKL)/osteoprotegerin (OPG) pathway. RANKL is a cytokine of the tumor necrosis factor (TNF) superfamily, expressed by osteoblasts and osteocytes. RANK is the receptor for RANKL, found on mature osteoclasts and osteoclast precursors. Upon RANKL binding to RANK, osteoclast differentiation is activated. OPG is another member of the TNF receptor family, secreted by osteoblasts, and acts as a soluble decoy receptor for RANKL preventing its binding to RANK, thereby impeding the osteoclast differentiation (Tantikanlayaporn et al. 2020). This balance can be interrupted by excessive activation of osteoclasts commonly seen in osteoporosis due to estrogen deficiency (Chen et al. 2021). Therefore, RANKL/RANK/OPG pathway plays a critical role in osteoporosis after menopause (Daniilopoulou et al. 2022).

Oxidative stress is a crucial pathogenic factor in osteoporosis development and progression (Munmun and Witt-Enderby 2021). RUNX2, Runt-related transcription factor 2, is essential for bone formation, reaching the maximal level in immature osteoblasts, and is downregulated in mature osteoblasts (Komori 2019). Therefore, targeting RUNX2 is crucial in osteoporosis management.

Anti-osteoporotic drugs cause serious adverse effects (Cheng et al. 2022a; Huang et al. 2022). Therefore, the demand of safer and more affordable alternatives replacing or minimizing the need for currently used pharmaceuticals is high.

Dipeptidyl peptidase-4 (DPP-4) inhibitors are an increasingly well-established oral antidiabetic drugs for the treatment of type 2 diabetic patients. Sitagliptin was the first oral Food and Drug administration (FDA)-approved agent introduced in this class (Kitaura et al. 2021). Studies have shown controversial data on the association between the use of DPP-4 inhibitors and fracture risk (Cheng et al. 2022b). Some studies showed that type 2 diabetic patients treated with DPP-4 inhibitors, including sitagliptin, have beneficial effects on the bone metabolism and reduced the fracture risk compared to placebo and other antidiabetic treatments probably via decreased inflammation-induced bone resorption, preventing cortical bone growth stagnation hence leading to increased femoral strength (Zhang et al. 2021). Others showed that treatment with DPP-4 inhibitors possibly increases the risk of bone fracture. Numerous preclinical studies speculated that certain antidiabetic medications perhaps have either worsening or repairing effects on bone quality (Nirwan and Vohora 2022). Thus, more studies are needed to evaluate the impact of DPP-4 inhibitors on postmenopausal osteoporosis. Moreover, the underlying molecular mechanisms of the effects of sitagliptin on bone protection are still unknown. Therefore, the aim of the current study is to investigate the possible anti-osteoporotic effect of sitagliptin in a rat model ovariectomized rats mimicking postmenopausal osteoporosis. Moreover, the role of oxidative stress, RANKL/OPG pathway, RUNX2, and apoptosis were investigated.

## Materials and methods

### Drugs

Sitagliptin tablets were purchased from Organon Pharma (Cramlington, UK).

### Chemicals

Catalase activity (CAT) and malondialdehyde (MDA) kits were purchased from Biodiagnostic Co. (Cairo, Egypt). OPG, RANKL and tartrate-resistant acid phosphatase (TRAP) ELISA kits were purchased from Cusabio Biotech (Newark, NJ, USA), Aviva Systems Biology (San Diego, CA, USA), and Elabscience Biotechnology Co., Ltd. (Wuhan, China), respectively. Calcium and phosphorus colorimetric assay kits were purchased from Bio-diagnostic Co. (Cairo, Egypt). The first-strand cDNA was synthesized using reverse transcription kit, and the used SYBR Green PCR master mix were purchased from Thermo Fisher Scientific Wilmington, DE, USA. The corresponding primer synthesis was entrusted to Invitrogen, Thermo Fisher Scientific, Inc. (Carlsbad, CA, USA). Primary antibody of rabbit anti-caspase-3 polyclonal antibody (ab13847) and goat anti- rabbit IgG H&L (HRP) (ab205718) were purchased from Abcam (Cambridge, UK). 3,3′-diaminobenzidine tetrahydrochloride was obtained from Sigma-Aldrich (Missouri, USA).

### Experimental animals

Twenty-four adult female Wistar rats weighing 230–250 g were purchased from the animal house National Research Center (Giza, Egypt). They were kept and housed in standard cages under proper environmental conditions throughout the time of the study: temperature (25 ± 3 °C), moisture (60 ± 10%), and regular light–dark cycles (every 12 h). Animals were fed on a standard rat pellet diet from United Co. for poultry production and indorsed free access to water for 2 weeks to adapt to the laboratory conditions.

### Study design

The rats were assigned randomly into four groups (*n* = 6): Group 1, sham-operated control (sham) rats underwent abdominal incision without ovariectomy; Group 2, ovariectomized (OVX) group; 2 months post- abdominal incision (Roch et al. 2020), the other two OVX groups (Groups 3 and 4) were treated orally with sitagliptin in doses of 10 and 20 mg/kg, respectively, for 4 weeks, except for the sham group; all groups underwent abdominal incision with bilateral ovariectomy to induce estrogen deficiency. All groups were anesthetized with thiopental sodium (25 mg/kg, i.p.) (Halici et al. 2008) before operation. Post-operative pain was reduced by buprenorphine (0.01–0.025 mg/kg, s.c.) (Mardas et al. 2021). The doses of sitagliptin treatment and duration of treatment were selected in accordance to prior studies (El-Agamy et al. 2016; Wang et al. 2017).

### Blood and bone samples

By the end of the experiment, rats were anesthetized, blood samples were withdrawn from the tail vein, centrifuged at 3000 rpm for 20 min, and the obtained sera were kept frozen at − 80 °C for evaluation of biochemical markers. After that, rats were sacrificed, and bilateral femurs and tibia were totally detached, cleaned off the adherent soft tissues, and washed with saline, then divided randomly into two sections: one for measuring bone mineral density (BMD) of femur and tibia by Dual-Energy X-ray Absorptiometry (DEXA), the other section was fixed in 10% formalin for histopathological analysis. The left femurs were removed and cleaned off adhering soft tissue; part was used for real time PCR analysis and the other was homogenized for estimation of biochemical analysis.

### Measurement of bone tissue oxidative stress

The catalase activity (CAT) and malondialdehyde (MDA) content were measured in bone homogenate of femurs using a commercial colorimetric kit according to the manufacturer’s protocol at 510 nm (Aebi 1984) and 534 nm (Ohkawa et al. 1979), respectively.

### Determination of bone turnover biomarkers

The concentrations of OPG, RANKL, and TRAP were evaluated in femur homogenate by using ELISA kits according to the manufacturers’ procedure.

### Determination of serum calcium (Ca) and phosphorus (P) levels

The serum levels of calcium and phosphorus in blood were assessed using colorimetric kits according to the manufacturer’s instructions at 585 nm (Gindler and King 1972) and 640 nm (El-Merzabani et al. 1977), respectively.

### Determination of femoral bone density

The soft tissue surrounding the proximal femur was removed. The bone mineral density (BMD) of femur was measured using DEXA (Osteosys Primus, Korea) at a scan pitch of 1.5 mm and a speed of 60 mm/s.

### RT-qPCR analysis

The mRNA expression of RUNX2 and protein kinase B (AKT) was assayed by RT-qPCR. Briefly, frozen left femur tissues, previously stored at − 80 °C, were placed into a mortar with liquid nitrogen and pulverized to a fine powder. The resulting bone powder (100 mg) was used for total RNA extraction using TRIzol method (Invitrogen, Thermo Fisher Scientific, Inc., Waltham, MA, USA) according to the manufacturer’s protocol. Isolated RNA was treated with DNAase kit (Invitrogen) to remove any residual DNA. The integrity and concentration of the isolated RNA were assessed by 1.0% agarose gel electrophoresis and Nanodrop ND-1000 spectrophotometer (Thermo Scientific, Wilmington, DE, USA), respectively. The first-strand cDNA was synthesized using Reverse Transcription kit. In a Light-Cycler system, the qPCR was performed with a SYBR Green PCR Master Mix, according to the manufacturer’s protocol. The condition of the qPCR amplification reaction was as follows: pre-denaturation at 95 °C for 10 s, and 40 cycles of 95 °C for 10 s and 58 °C for 20 s, and 72 °C for 15 s with a final extension (95 °C for 5 min) to complete the amplification procedure. The relative mRNA expression was calculated by using comparative (2^−ΔΔCT^) method, and the results are presented as fold-change respective to the normal control mean values, normalized to β-actin (Sabry et al. 2023). Each sample was analyzed with three replicates. The corresponding primer synthesis was entrusted to Invitrogen Co., Ltd., and the primer sequences are shown in Table 1.

**Table 1:** *RT*-*qPCR* primer sequences for target genes

Gene	Forward (5′−3′)	Reverse (5′−3′)	Accession #
Runx2	GCGTCCTATCAGTTCCCAAT	ATCAGCGTCAACACCATCAT	XM_017596552.2
AKT-1	TCGTGTGGCAAGATGTGTATGAGA	CAGGCGGCGTGATGGTGAT	XM_008764918.2
β-actin	GGTGGGTATGGGTCAG	ATGCCGTGTTCAATGG	NM_031144.3

### Histopathological examination

The right femur bones were collected from different groups and fixed in 10% neutral buffered formalin for 48 h then decalcified in 20% EDTA (Sigma) for 8 weeks. The femur bones were processed for obtaining 3–4 µm paraffin embedded sections. The sections were stained with hematoxylin and eosin stain (H & E). Others paraffin embedded tissue sections were deparaffinized and rehydrated then stained with Alizarin Red stain following the methods described by Fouad-Elhady et al. (2020) and Masson’s trichrome-staining according to Shehata et al. (2017). The intensity of red color in Alizarin Red stained sections and the blue color in Masson’s trichrome-stained sections were measured using Image J analysis software (NIH, Bethesda, MD, USA). The tissue sections from all groups were examined under a light microscope at different magnification powers. The mean cortical bone thickness (Um), trabecular bone thickness, and the number of osteoblasts and osteoclasts per high power field (HPF) were recorded from 7 non-overlapping fields of each specimen using Image J analysis software (NIH, Bethesda, MD, USA).

### Immunohistochemical analysis of caspase-3

Caspase-3 immunohistochemical analysis was done according to El-Marasy et al. (2019). Deparaffinization and rehydration of the tissue sections were performed. Antigenic retrieval of the epitopes was done according to methods described by Abu‐Elala et al. (2015). The tissue sections were incubated with primary antibody of rabbit anti-Caspase-3 polyclonal antibody (ab13847) at 1:100 dilution for overnight in a humidified chamber. The tissue sections were incubated with drops of Hydrogen Peroxide Blocking solution to block the endogenous peroxidase activity, then the sections incubated with goat anti- rabbit IgG H&L (HRP) (ab205718). The slides were incubated with 3,3′-diaminobenzidine tetrahydrochloride (DAB) for 10 min and counterstained with Mayer’s hematoxylin and mounted. The percentage of active Caspase-3 immunopositive stained cells (%) was determined in shaft bone by counting the number of positive chondrocytes and total number of cells. The image analyses of the stained sections were done by Image J program (NIH, Bethesda, MD, USA).

### Statistical analysis

One-way analysis of variance (ANOVA) followed by Tukey’s multiple comparison test for post hoc comparisons between groups was performed using GraphPad Prism Program, San Diego, USA; version 8; *p* < 0.05. The values were expressed as mean ± SEM, and values less than 0.05 are considered significant. Mann–Whitney analysis was used to examine data from histopathological and immunohistopathological exams. Graphs are presented as Box and whisker plots.

## Results

### Sitagliptin reduced malondialdehyde content and elevated catalase content

**Fig. 1:**
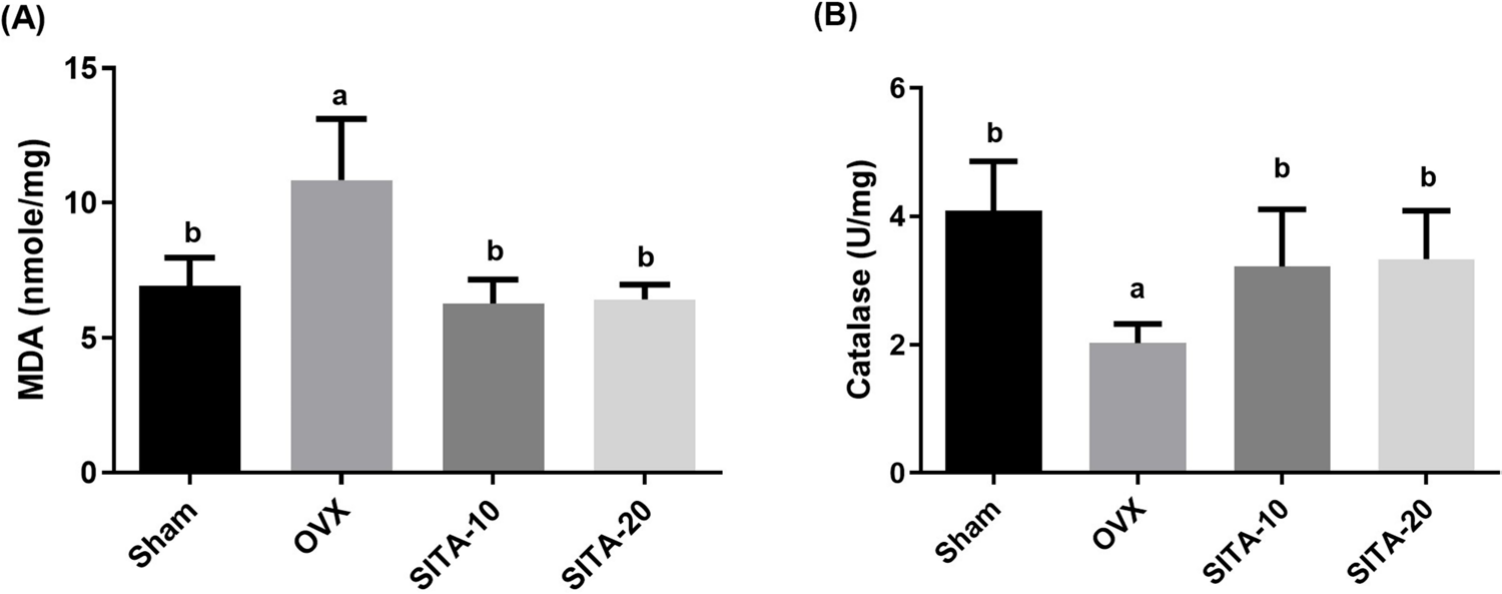
Effect of sitagliptin on **A** malondialdehyde and **B** catalase. Each bar represents the mean ± SEM of six rats in each group. ^a^vs sham rats at* p* ≤ 0.05, ^b^vs OXV control rats at *p* ≤ 0.05. SITA-10, sitagliptin 10 mg/kg; SITA-20,sitagliptin 20 mg/kg

As depicted in Fig. 1A, the bone oxidative stress marker, malondialdehyde (MDA), was shown to be markedly elevated in OVX control rats to 1.56 fold (*F* = 15.32; *p* = 0.0001) as compared to the sham group. However, in Fig. 1B, catalase (CAT) activity was significantly decreased in OVX control group to 0.50 fold (*F* = 8.53; *p* = 0.0004) as compared to the sham. Sitagliptin (SITA) at 10 and 20 mg/kg noticeably reduced the bone MDA levels to 0.58 fold (*F* = 15.32; *p* < 0.0001) and 0.59 fold (*F* = 15.32; *p* < 0.0001), respectively, compared to the OVX group. Bone catalase (CAT) activity was significantly increased in SITA (10, 20 mg/kg)-treated rats, to 1.59 fold (*F* = 8.53; *p* = 0.042) and 1.64 fold (*F* = 8.53; *p* = 0.024) as compared to the OVX control rats.

### Sitagliptin downregulated RANKL, OPG, and TRAP content

The levels of bone turnover regulators, osteoprotegerin (OPG), and receptor activator of NF-κB ligand (RANKL) were assessed in femoral homogenate of rats. The activity of TRAP, an osteoclast activity indicator was also assessed. OPG exhibited significant decrease in bone homogenate of OVX control rats to 0.68 fold (*F* = 16.88; *p* = 0.0004), as compared to sham rats (Table 2). Though, bone RANKL content was significantly elevated in bone homogenate of OVX control rats to 1.47 fold (*F* = 8.28;* p* = 0.0141), as compared to sham rats. TRAP activity in bone homogenate was remarkably elevated in OVX rats to 1.51 fold (*F* = 17.17; *p* = 0.0002), as compared to sham rats. Bone OPG levels were significantly elevated in SITA (10 and 20 mg/kg)-treated rats, to 1.55 fold (*F* = 16.88; *p* < 0.0001) and 1.59 fold (*F* = 16.88; *p* < 0.0001), respectively as compared to OVX control rats. However, RANKL bone contents and the activities of TRAP were significantly decreased in SITA-10 to 0.59 fold (*F* = 8.28; *p* = 0.0017) and 0.61fold (*F* = 17.17; *p* < 0.0001), respectively and SITA −20 groups to 0.6 fold (*F* = 8.28; *p* = 0.0022) and 0.62 fold (*F* = 17.17; *p* < 0.0001), respectively, as compared to OVX control group. Remarkably, RANKL/OPG ratio (0.475) was increased significantly in femur tissue homogenate of OVX rats compared to sham rats (0.220). SITA-treated rats revealed lowered RANKL/OPG ratio compared to OVX control rats (SITA-10; 0.180 and SITA-20; 0.179).

**Table 2:** Effect on bone turnover biomarkers

Groups	Parameters		
	OPG (pg/mg)	RANKL (pg/mg)	TRAP (ng/mg)
Sham	747.4 ^b^ ± 35.89	164.4 ^b^ ± 7.9	3.7 ^b^ ± 0.24
OVX	510.0 ^a^ ± 20.07	242.1 ^a^ ± 30.27	5.6 ^a^ ± 0.38
SITA-10	792.6 ^b^ ± 33.18	142.9 ^b^ ± 5.14	3.4 ^b^ ± 0.16
SITA-20	811.6 ^b^ ± 42.73	145.4 ^b^ ± 6.61	3.5 ^b^ ± 0.17

Values represent mean ± SEM of six rats in each group. ^a^vs sham rats at* p* ≤ 0.05. ^b^vs OXV control rats at *p* ≤ 0.05. SITA-10: sitagliptin 10 mg/kg; SITA-20: sitagliptin 20 mg/kg. OPG: osteoprotegerin; RANKL: receptor activator of NF-κB ligand; TRAP: tartrate-resistant acid phosphatase

#### Sitagliptin elevated serum calcium (Ca) and phosphorus (P) levels

Serum calcium (Ca) level was significantly declined to 0.29 fold (*F* = 18.16; *p* < 0.0001) in OVX control group, compared to the sham operated rats (Table 3). However, serum Ca levels were significantly increased in SITA-treated groups at levels 10 mg/kg and 20 mg/kg to 2.41 fold (*F* = 18.16; *p* = 0.0027) and 2.14 fold (*F* = 18.16;* p* = 0.0158) and, respectively, compared to OVX control rats. On contrary, serum phosphorus (P) levels were significantly declined in OVX control group to 0.79 fold (*F* = 409.1; *p* < 0.0001), compared to the sham operated rats, while serum P levels were significantly increased in SITA-treated groups at 10 mg/kg to 1.08 fold (*F* = 409.1; *p* < 0.0001), compared to OVX control rats.

**Table 3:** Effect on calcium (Ca) and phosphorus (P) serum levels

Groups	Parameters	
	Serum calcium (Ca)	Serum phosphorus (P)
Sham	9.90 ^b^ ± 1.02	12.41 ^b^ ± 0.06
OVX	2.83 ^a^ ± 0.41	9.77 ^a^ ± 0.01
SITA-10	6.81 ^b^ ± 0.60	10.58 ^b^ ± 0.13
SITA-20	6.05 ^b^ ± 0.54	8.19 ^b^ ± 0.10

Values represent mean ± SEM of six rats in each group. ^a^vs sham rats at* p* ≤ 0.05. ^b^vs OXV control rats at *p* ≤ 0.05. SITA-10: sitagliptin 10 mg/kg; SITA-20: sitagliptin 20 mg/kg

#### Sitagliptin upregulates mRNA expression of AKT and RUNX2

To determine the effect of sitagliptin on osteoblastic cell differentiation, the expression levels of Akt/RUNX2-related genes, AKT and its downstream RUNX2, were evaluated by qRT-PCR. The analysis revealed that mRNA expression of AKT and RUNX2 decreased 0.4-fold (*F* = 142.2; *p* < 0.0001) and 0.4-fold (*F* = 80.21; *p* < 0.0001), respectively, in OVX group compared with sham group (Table 4). Interestingly, treated groups showed moderate increase in the expression of these genes AKT and RUNX2 to 0.5 fold (*F* = 142.2; *p* = 0.0016) and 0.6 fold (*F* = 80.21; *p* < 0.0001) in SITA-10-treated group, respectively, and 0.6 fold (*F* = 142.2; *p* < 0.0001) and 0.8 fold (*F* = 80.21; *p* < 0.0001) in SITA-20-treated group, respectively, in comparison to sham rats (Table 4).

**Table 4:** Effect on bone Run-X2 and AKT relative mRNA expression level

Groups	Parameters	
	Relative expression levels (Fold changes from Sham level)	
	RUN-X2	AKT
Sham	1.0 ^b^ ± 0	1.0 ^b^ ± 0
OVX	0.4 ^a^ ± 0.04	0.4 ^a^ ± 0.03
SITA-10	0.6 ^ab^ ± 0.04	0.5 ^ab^ ± 0.02
SITA-20	0.8 ^ab^ ± 0.03	0.6 ^ab^ ± 0.02

Values represent mean ± SEM of six rats in each group. ^a^vs sham rats at* p* ≤ 0.05. ^b^vs OXV control rats at *p* ≤ 0.05. SITA-10: sitagliptin 10 mg/kg; SITA-20: sitagliptin 20 mg/kg. RUNX2: runt-related transcription factor 2; AKT: protein kinase B

#### Sitagliptin upregulated femoral and tibia bone density

**Fig. 2:**
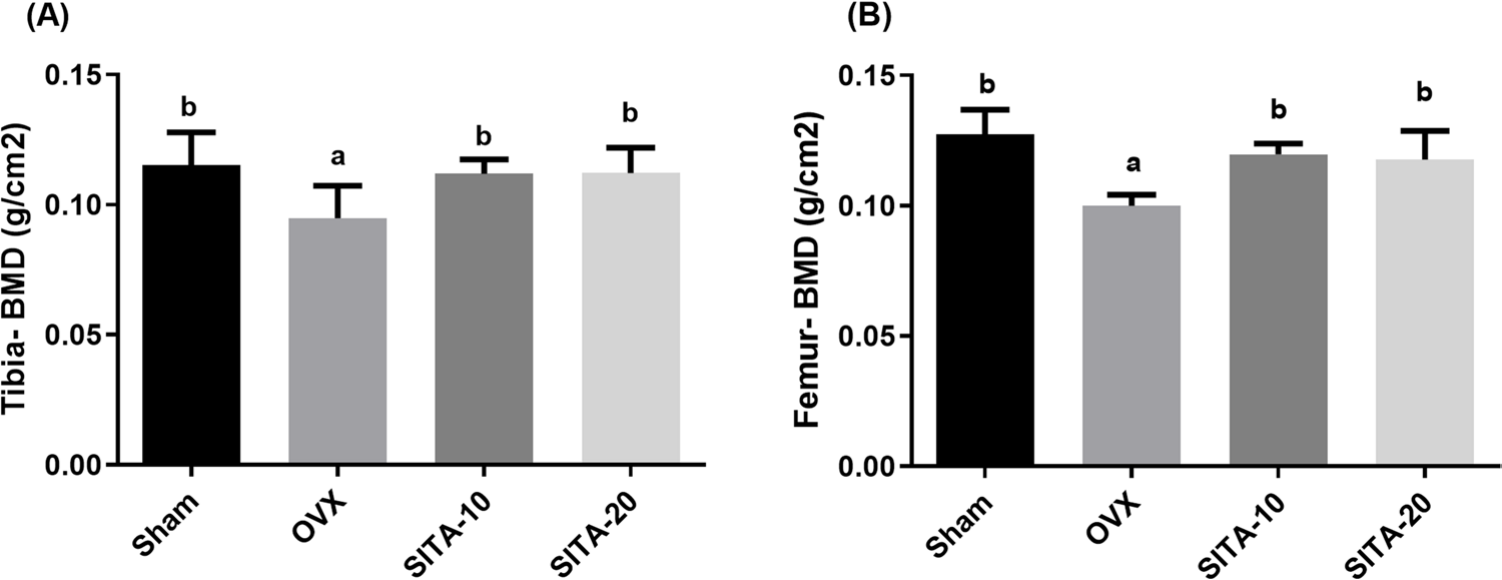
Effect sitagliptin bone mineral density of **A** tibia and **B** femur. Each bar represents the mean ± SEM of six rats in each group. ^a^vs sham rats at* p* ≤ 0.05, ^b^vs OXV control rats at *p* ≤ 0.05. SITA-10, sitagliptin 10 mg/kg; SITA-20, sitagliptin 20 mg/kg

Bone mineral density (BMD) of both tibia and femur were measured by DEXA. Figure 2 demonstrates that ovariectomy significantly reduced BMD of both tibia to 0.75 fold (*F* = 4.66; *p* = 0.0154) as shown in Fig. 2A and femur to 0.77 fold (*F* = 13.25; *p* < 0.0001) as in Fig. 2B compared to sham operated rats. SITA-10 and SITA-20 significantly elevated the BMD of tibia to 1.22 fold (*F* = 4.66; *p* = 0.047) and 1.22 fold (*F* = 4.66; *p* = 0.044), respectively, and femur to 0.92 fold (*F* = 13.25; *p* = 0.0016) and 0.92 (*F* = 13.25; *p* = 0.0044), respectively, compared to the OVX control rats.

#### Sitagliptin attenuated histopathological alterations

**Fig. 3:**
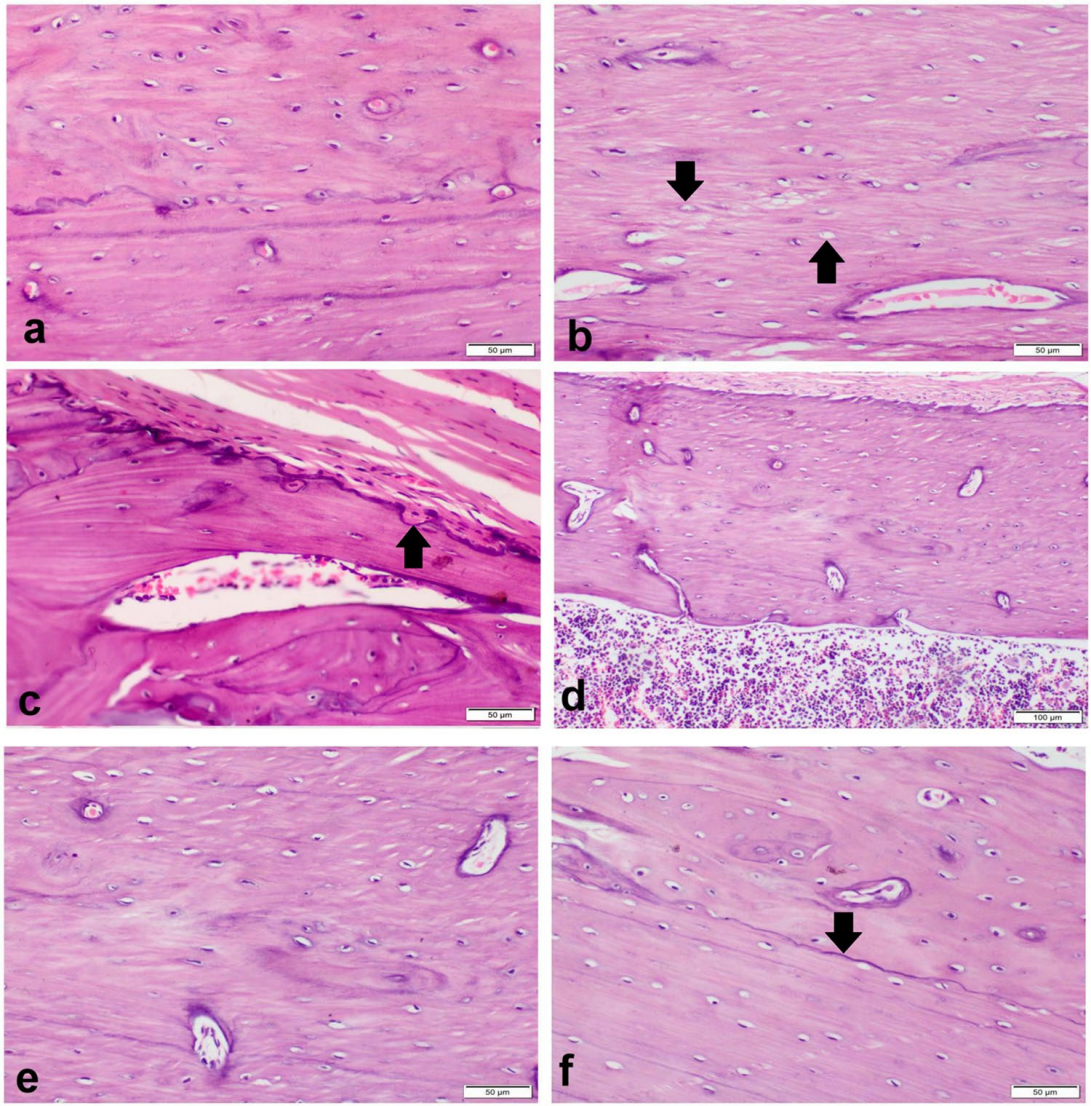
Photomicrograph of the bone shaft of the femur with H&E-stain. **a** Sham group showing normal architecture compact cortical bone. **b** OVX control group showing degenerated osteocytes with multiple empty lacunae (arrow). **c** OVX control group showing erosive endosteal surface (arrow). **d**, **e** SITA-10 showing a nearly normal compact bone. **f** SITA-20 showing a basophilic cement line (arrow) separating the borders of old and new bone matrix. SITA-10, sitagliptin 10 mg/kg; SITA-20, sitagliptin 20 mg/kg

**Fig. 4:**
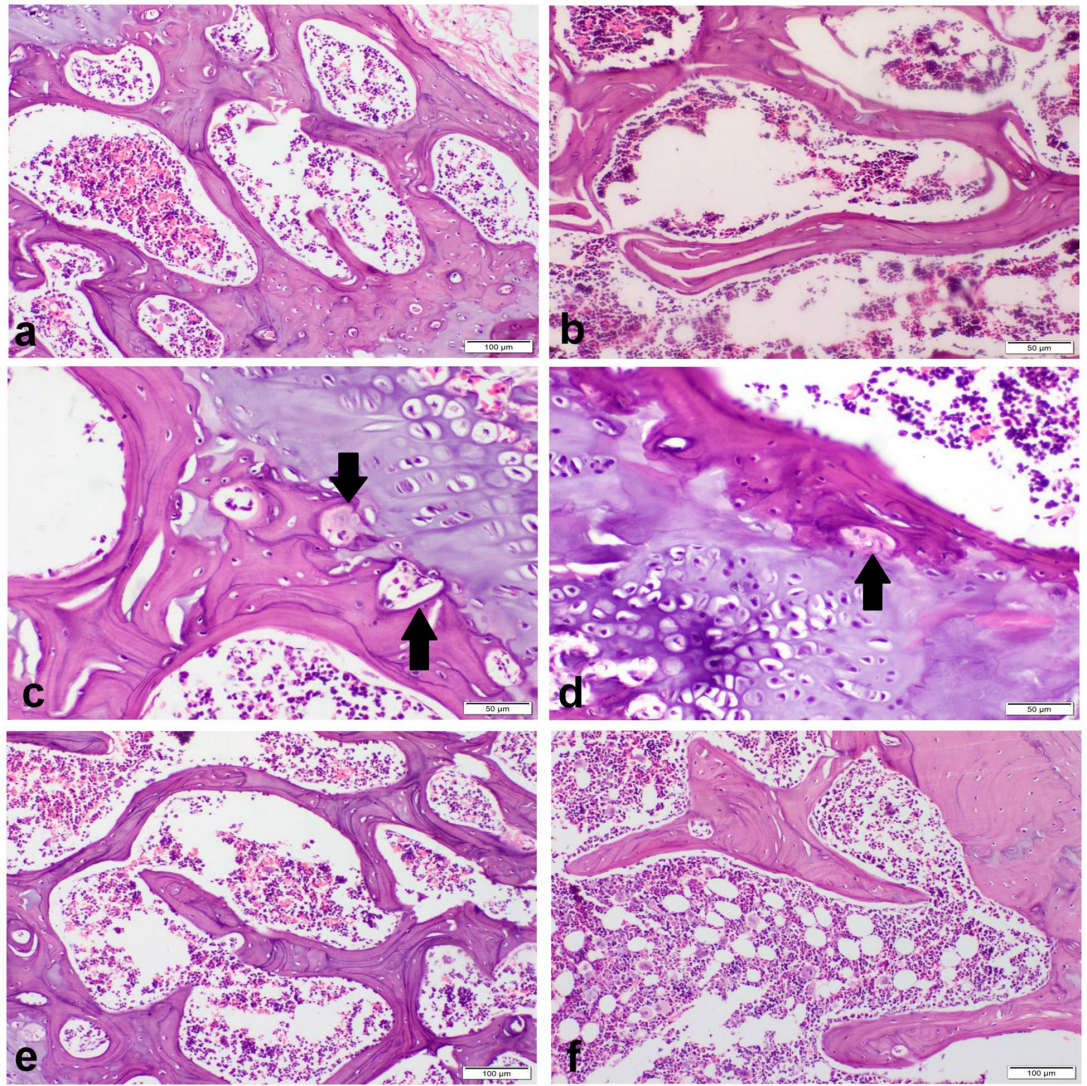
Photomicrograph of cancellous bone of the femur with H&E-stain. **a** Sham group showing normal a network of bony trabeculae containing bone marrow. **b** OVX control group showing thin trabecular bones with marked widening of interconnecting spaces. **c** OVX control group showing erosive cavities containing osteoclasts (arrows). **d**, **e** SITA-10 normal osteocytes with regular and smooth endosteal surface. **f** SITA-20 showing normal cancellous bone. SITA-10, sitagliptin 10 mg/kg; SITA-20, sitagliptin 20 mg/kg

The sham group showed normal compact cortical bone with regular and smooth outer and inner circumferential lamellae. The interstitial bone lamellae contained normally organized osteocytes embedded in their lacunae and normal Haversian canals containing blood vessels, nerves, and lymphatics (Fig. 3a). Periosteum was consisted of a thick outer fibrous layer. The endosteum had a smooth surface and lined by osteoprogenitor cells, osteoblasts, and osteoclasts. The osteoblast cells were single-nucleated, rod-shaped cells attached to bone surface. The inner cancellous bones of femur metaphysis composed of a network of bony trabeculae with interconnecting spaces containing bone marrow (Fig. 4a). OVX control group showed marked degenerated compact bone with irregular inner circumferential lamellae that appeared with multiple notches filled with osteoclasts which appeared with eosinophilic cytoplasm and multiple nuclei. Osteocytes were degenerated and necrosed with multiple empty lacunae (Fig. 3b). The trabecular bones appeared thin with marked widening of interconnecting spaces (Fig. 4b). There were numerous erosive cavities containing osteoclasts (Fig. 4c). The groups treated with sitagliptin revealed a nearly normal compact (Fig. 3d, e) and trabecular bones with many blood vessels; most of osteocytes were normal with few necrosed one, and the endosteal surface was regular and smooth (Fig. 4d–f). There was a basophilic cement line separating the borders of old and new bone matrix (Fig. 3f).

#### Sitagliptin ameliorated morphometrical alterations of femur

**Fig. 5:**
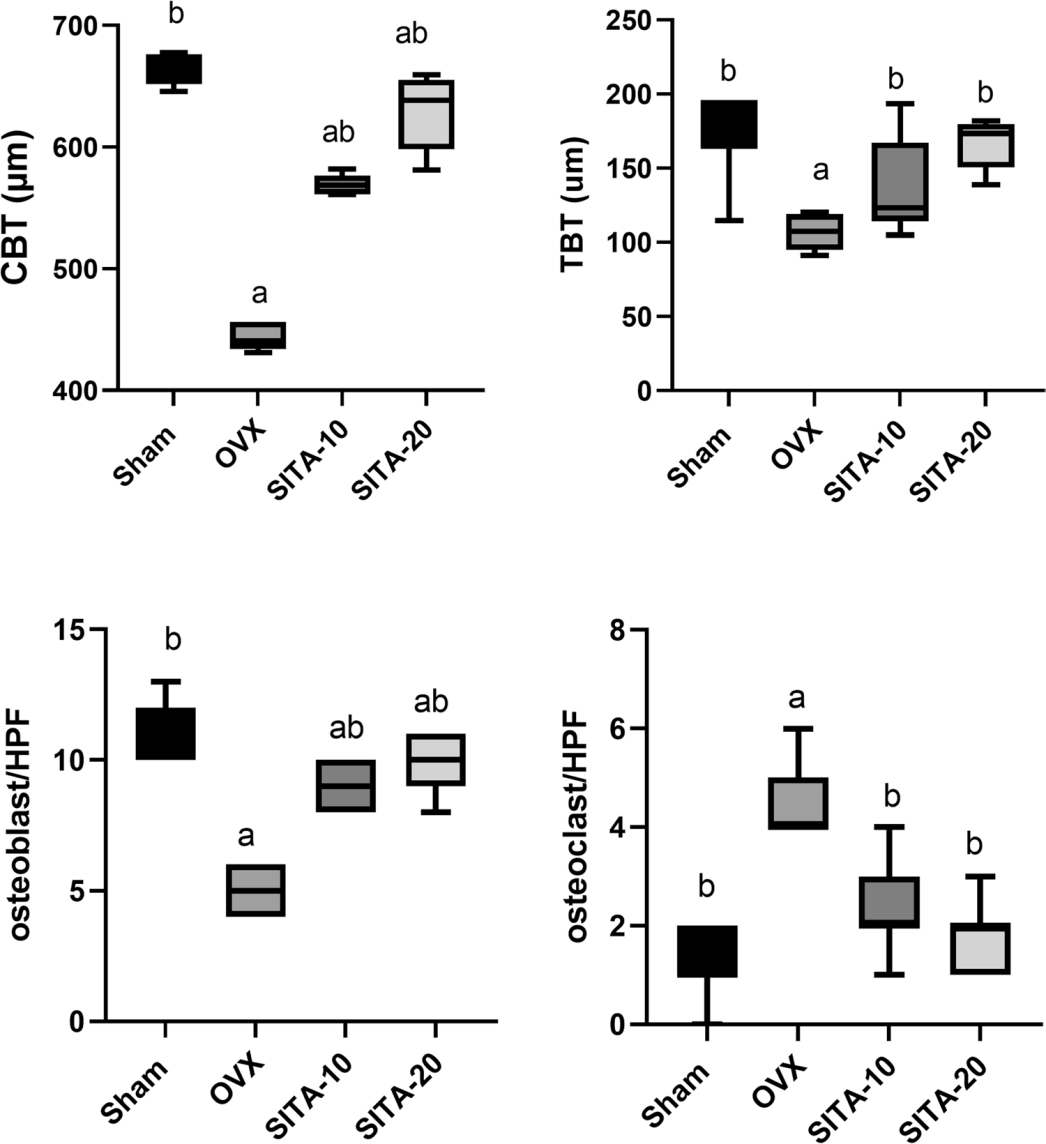
Morphometrical analysis of femur bone. **A** Cortical bone thickness. **B** Trabecular bone thickness. **C** Osteoblasts number. **D** Osteoclast number. Each bar represents median. ^a^vs sham rats at* p* ≤ 0.05, ^b^vs OVX control rats at *p* ≤ 0.05. SITA-10, sitagliptin 10 mg/kg; SITA-20, sitagliptin 20 mg/kg

The cortical bone thickness (CBT), trabecular bone thickness (TBT), and osteoblasts measurements showed a significance reduction in OVX control group compared to sham group and a significance elevation in groups treated with sitagliptin when compared to osteoporotic control group (Fig. 5a, b, c). However, the osteoclasts number was significantly increased in osteoporotic control group comparing to sham group and significantly decreased in treated groups with sitagliptin comparing to OVX control group (Fig. 5d).

#### Sitagliptin reduced bone decalcification using Alizarin red S staining

**Fig. 6:**
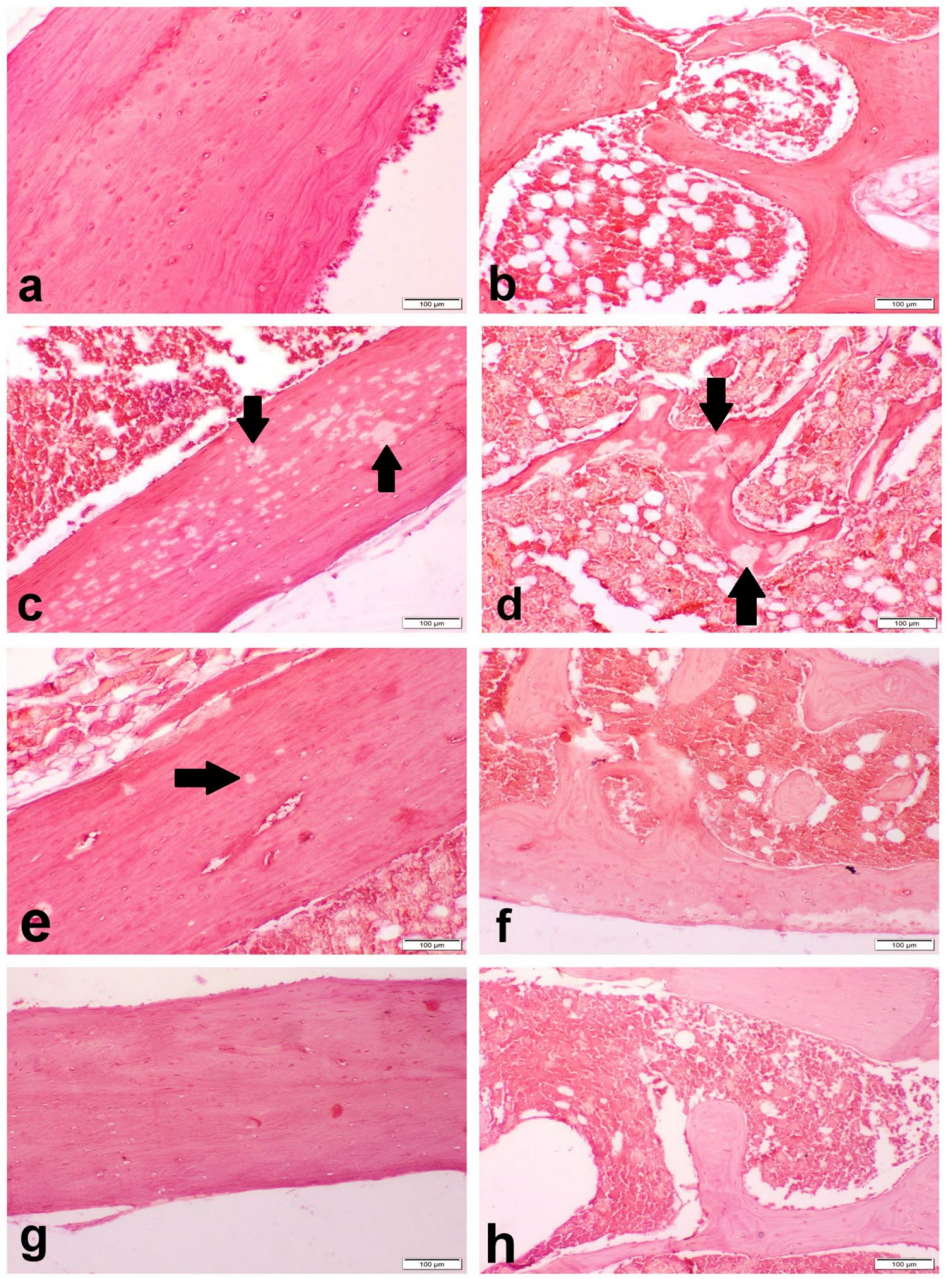
Photomicrograph of Alizarin red-stained tissue sections of the bone shaft and cancellous of the femur. **a**, **b** Sham group showing homogenous red staining calcified bone. **c**, **d** OVX control group showing multiple area of faint red decalcified bone tissues (arrow). **e**, **f** SITA-10 mg/kg showing few areas of decalcified bones (arrow). **g**, **h** SITA-20 mg/kg showing homogenous red calcified bone. SITA-10, sitagliptin 10 mg/kg; SITA-20, sitagliptin 20 mg/kg

As depicted in Fig. 6, the sham group showed homogenous red staining calcified bone (Fig. 6a, b). OVX control group exhibited multiple areas of decalcified bone tissues (Fig. 6c, d). Sitagliptin-treated groups revealed red staining calcified bone with very small area of decalcified bone tissues (Fig. 6e–h).

#### Sitagliptin increased collagen fiber using Masson’s trichrome-staining

**Fig. 7:**
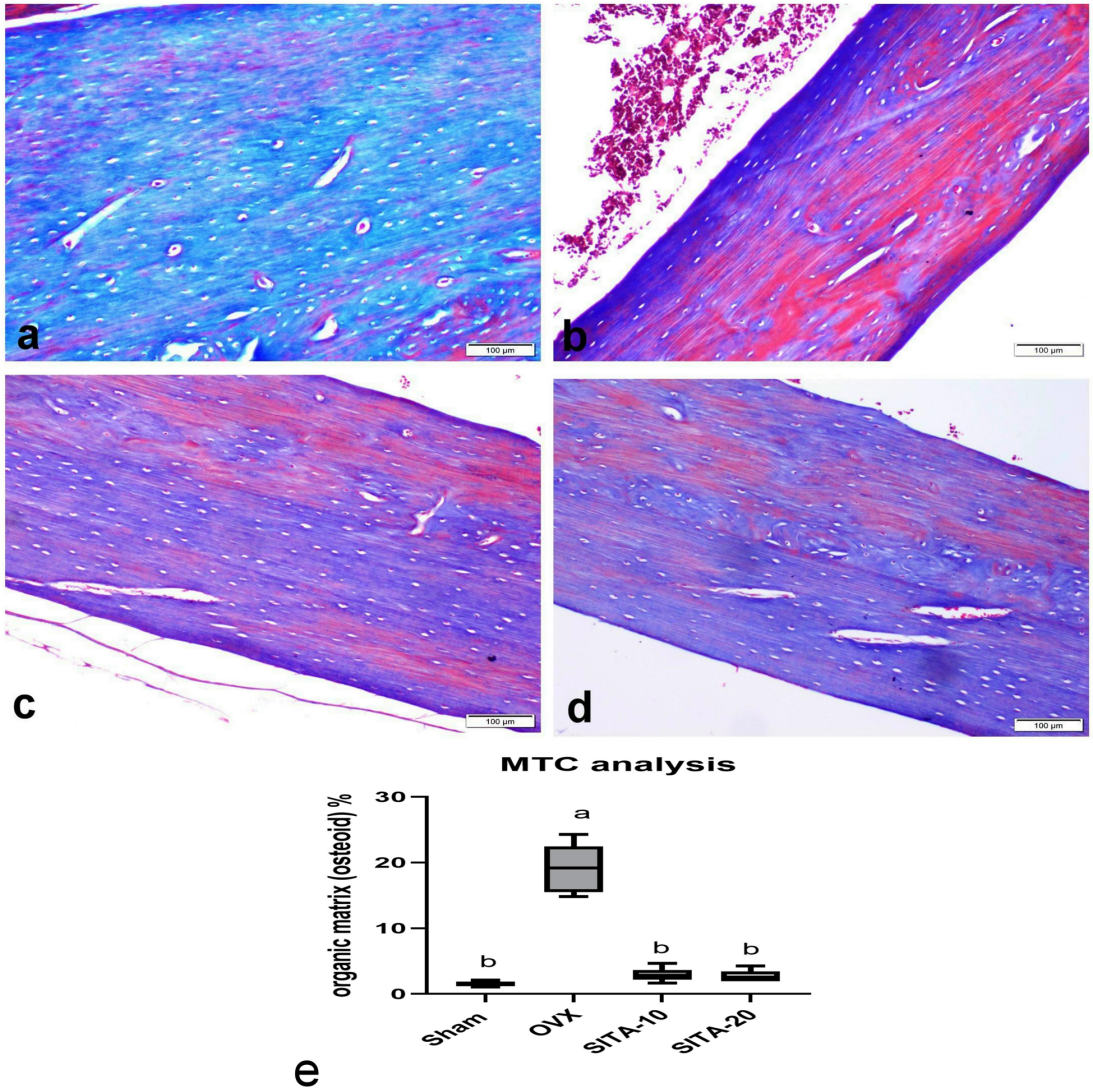
Photomicrograph of MT-stained tissue sections of the bone shaft of the femur. **a** Sham group showing regular lamellar arrangement of blue-stained collagen fiber. **b** OVX control group showing marked reduction in the blue-stained collagen fiber with increase of red-stained osteoid tissue. **c** Sitagliptin 10 mg/kg and **d** sitagliptin 20 mg/kg showing marked increase in blue-stained collagen fiber. **e** Bar chart represents organic matrix (osteoid) area %. Values are expressed as median ^a^vs sham rats at* p* ≤ 0.05, ^b^vs OXV control rats at *p* ≤ 0.05. SITA-10, sitagliptin 10 mg/kg; SITA-20, sitagliptin 20 mg/kg

The sham group showed regular lamellar arrangement of blue-stained collagen fiber using Masson’s trichrome-staining (MT) (Fig. 7a). Osteoporotic control group revealed a significant reduction in the blue-stained collagen fiber with a loss of its lamellar arrangement and a significant increase red-stained bone organic matrix (osteoid) (Fig. 7b). Groups treated with sitagliptin showed significant increase in blue-stained collagen fiber when compared with OVX control group (Fig. 7c, d, e).

#### Sitagliptin reduced caspase-3 protein expression

**Fig. 8:**
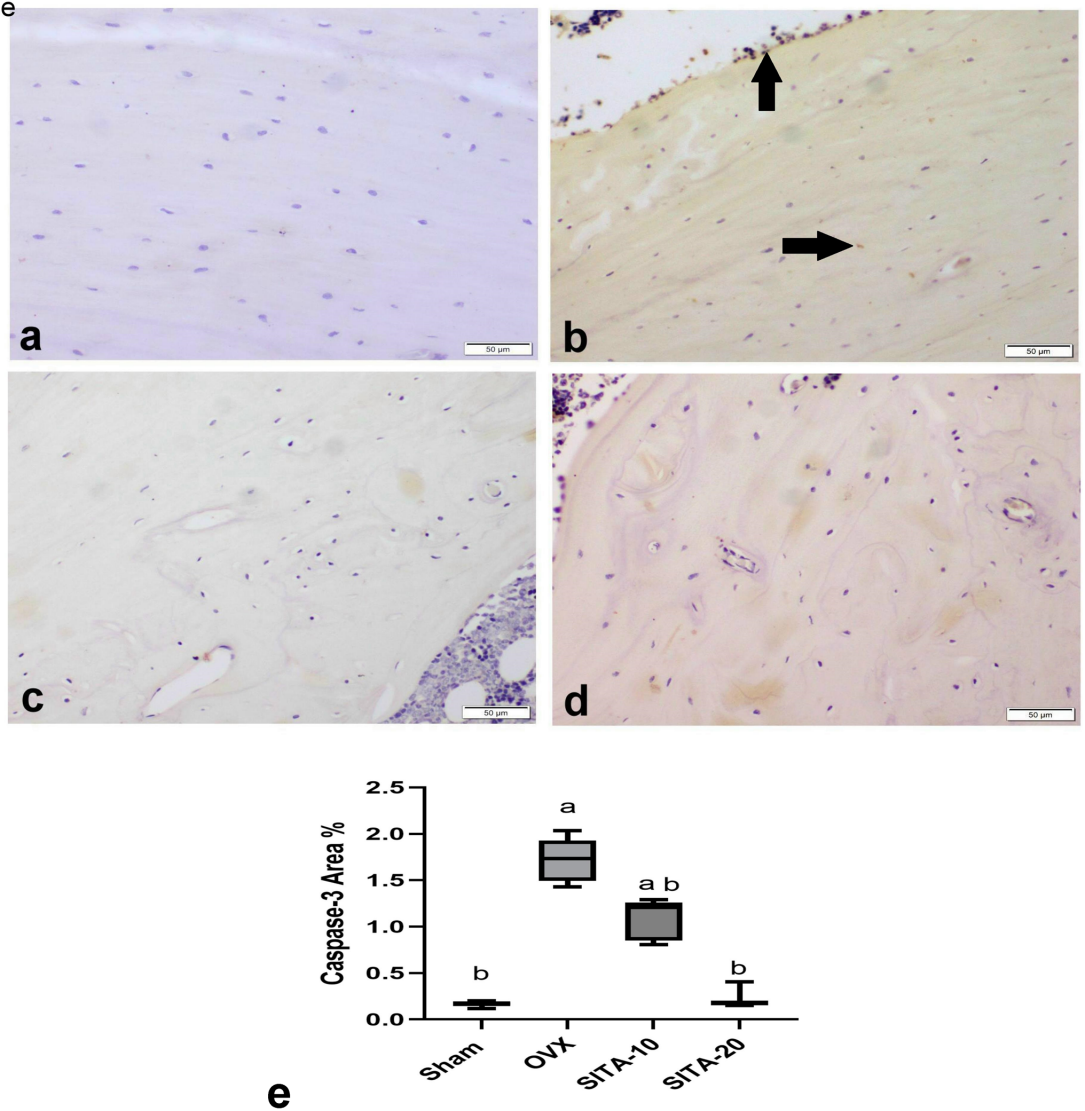
Photomicrograph of Caspase-3 immunohistochemistry in shaft bone of the femur. **a** Sham group. **b** OVX control group showing Caspase-3 immune-positive cells (arrow). **c** SITA 10 mg/kg. **d** SITA 20 mg/kg. **e** Bar chart represents Caspase-3 immune-stained cells %. Values are expressed as median ^a^vs sham rats at* p* ≤ 0.05, ^b^vs OXV control rats at *p* ≤ 0.05. SITA-10, sitagliptin 10 mg/kg; SITA-20, sitagliptin 20 mg/kg

Figure 8 summarizes the results of immunohistochemical analyses of active caspase-3 expression in the different experimental groups. The percentage of caspase-3 immune-positive cells was significantly increased in osteoporotic control groups than the sham group and significantly decreased in sitagliptin treated groups comparing to OVX control group.

## Discussion

The current study discloses novel mechanistic insights on the protective role sitagliptin (10 and 20 mg/kg) on OVX-induced osteoporosis in rats.

In this study, OVX-induced osteoporosis model in rats was used as it closely resembles postmenopausal women suffering from osteoporosis. The model was successively established evidenced by disruption in levels of bone metabolic markers: Ca, P as well as BMD and histopathological alterations.

OVX group revealed degenerated compact bone with presence of multiple notches filled with osteoclasts, degenerated osteocytes, increased number of osteoclasts, and decreased number of osteoblasts indicating increase of bone resorption. This implies that osteoclastic bone resorption outweighs the anabolic activity of osteoblasts. The deficiency of estrogen is the major factor that speeding up bone remodeling (Grassi et al. 2006; Saleh and Saleh 2011). Subsequently, CBT and TBT were significantly decline in OVX rats. The present study reported a significant decline in collagen fibers in bone shaft of OVX group. This finding was previously observed in many studies; OVX results in significant bone loss and degradation of collagen orientation, leading to the presentation of brittle bone and a reduction of bone toughness (Ozasa et al. 2019; Sato et al. 2021).

Decreased amount of collagenous fiber together with increased bone resorption resulted in the elevation in the incidence of bone fracture in osteoporosis (Neumann et al. 2016; Starup-Linde and Vestergaard 2016).

Sitagliptin alleviated bone metabolism as it restored serum Ca and P levels and thereby ameliorated bone-related disorders. Impairment in calcium balance mimics menopause due to estrogen deficiency that results in high urinary Ca excretion (Saleh et al. 2020; Gholami et al. 2022). Moreover, hypophosphatemia resulting from OVX also leads to bone loss (Liu et al. 2022).

BMD is a crucial criterion for assessment of osteoporosis (Neri et al. 2023). Sitagliptin also ameliorated BMD of rats’ femur and bone morphometric changes as it alleviated osteoclastic resorption marked by reduction in osteoclast number as well as CBT and TBT thickness. Moreover, sitagliptin enhanced bone formation and elevated number of osteoblasts. Those findings show that sitagliptin improved bone strength and architecture. In line with our findings, it has been reported that sitagliptin improved BMD at the lumbar spine in OVX rats and enhanced bone mass and biomechanics in diabetic rats (Cusick et al. 2013; Glorie et al. 2014). In the same manner, sitagliptin ameliorated bone strength via reducing the osteoclast number and increasing the osteoblast number in mice fed with high-fat diet (Mansur et al. 2019). Moreover, prior studies have demonstrated that attenuation of bone metabolism by restoring Ca and P was accompanied by increment in BMD and that Ca supplementation elevates BMD (Mao et al. 2021; Wang et al. 2021); thereby, it can be deduced that sitagliptin restored BMD by alleviating bone metabolism.

Sitagliptin significantly increased bone catalase (CAT) activity and reduced lipid peroxidation evidenced by reduction in MDA femur bone content and augmented anti-oxidant defence as it restored catalase anti-oxidant activity, revealing that sitagliptin combated osteoporosis via its anti-oxidant activity.

Oxidative stress diminishes osteoblast proliferation and differentiation, thus provoke the development of osteoporosis (Wang et al. 2022). In this study, sitagliptin elevated RUNX2 gene expression, a crucial osteogenic factor, and a vital modulator in bone formation and osteoblast differentiation. Thus, sitagliptin enhanced osteoblast differentiation in OVX rats via its antioxidant activity. In accordance, dipeptidyl peptidase 4 (DDP-4) inhibitors as anagliptin promoted osteoblastic differentiation via elevation of RUNX2 osteogenic factor of MC3 T3-E1 pre-osteoblastic cells (Dong et al. 2020).

In this study, sitagliptin supressed RANKL, stimulated OPG femur bone contents, and reduced RANKL/OPG ratio revealing that sitagliptin repressed RANKL enhancement of osteoclastic differentiation, osteoclastogenesis, and augmented osteogenesis via suppression of RANKL/OPG pathway.

While OPG interacts with RANKL and prevents its binding to the receptor, RANKL attaches to its receptor RANK on osteoclasts and stimulates bone resorption. High RANKL/OPG ratio in osteoporosis indicates decline in bone formation and enhanced bone destruction (Jiang et al. 2023). It is well established that overproduction of reactive oxygen species (ROS) promotes osteoclast differentiation, bone resorption, and thereby bone remodelling (Wong et al. 2022). Therefore, it can be deduced that sitagliptin downregulated RANKL/OPG pathway through its anti-oxidant activity. In accordance with the current finding, prior study showed that sitagliptin ROS scavenging activity inhibited RANKL-induced osteoclastogenesis in vitro (Wang et al. 2017).

RANKL/RANK binding on osteoclast precursors stimulate the elevation of the osteoclastogenesis key indicator, TRAP, which is an essential enzyme involved in bone mineralization and skeletal development. TRAP is primarily produced by osteoclasts. According to Tolba et al. (2017), TRAP is a crucial marker of osteoclast activity and quantity, and elevated levels of it are linked to osteoporosis and other bone diseases. AKT phosphorylation and activation promotes osteclast differentiation and survival. In this study, it is obvious that sitagliptin restrained osteoclastogenesis as it reduced TRAP content and reduced AKT activation and elevated its gene expression in femur bone. In accordance, sitagliptin counteracted RANKL- induced AKT phosphorylation and activation and thereby osteoclastogenesis in OVX mouse model (Wang et al. 2017).

Previous studies demonstrated that treatments of osteoporosis elevated RUNX2 the osteogenic factor and reduced RANKL/OPG ratio (Tolba et al. 2017; Arafa et al. 2023); therefore, the relationship between RUNX2 and RANKL/OPG is indirectly proportional. However, activation and phosphorylation of AKT is a downstream of RANKL/OPG pathway in osteoclastogenesis (Tolba et al. 2017). Therefore, AKT activation and phosphorylation is directly proportional to the activation of RANKL/OPG pathway.

Sitagliptin’s anti-apoptotic effect on osteoblast may be partly mediated by its anti-oxidant effect, as it is well- documented that oxidative stress combats osteoblastic differentiation, proliferation, and thereby provoke apoptosis of osteoblast (Ma et al. 2020).

## Conclusions

In conclusion, sitagliptin showed novel protective effect against OVX-induced osteoporosis in rats via combating osteoclastogenesis and promoting osteoblast protection. Sitagliptin’s anti-oxidant effect is closely involved in its upregulation of RUNX2, downregulation of RANKL/OPG, TRAP, AKT pathways, and protection against osteoblast apoptosis.

## Limitation of the study

A limitation of the study is that more doses of sitagliptin are needed to be investigated to determine the optimal dose of sitagliptin that produced anti-osteoporotic effect. More bone metabolism markers need to be investigated to be able to correlate the relation of alterations in bone density, variations in bone metabolism markers, and the diagnosis and assessment of treatment effectiveness. Also, bone morphometric proteins need to be measured. Also, future studies are still warranted to explore the other molecular mechanisms of sitagliptin involved in its protective effect in osteoporosis-associated bone diseases.

## Data Availability

Data will be available upon request.

## Abbreviations

*AKT*: Protein kinase B
*ANOVA*: One-way analysis of variance
*BMD*: Bone mineral density
*BMPs*: Bone morphogenetic proteins
*Ca*: Calcium
*CAT*: Catalase
*CBT*: Cortical bone thickness (CBT),
*DEXA*: Dual-Energy X-ray Absorptiometry
*DPP-4*: Dipeptidyl peptidase-4
*FDA*: Food and Drug Administration
*GLP-1*: Glucagon-like peptide-1
*MDA*: Malondialdehyde
*MT*: Masson’s trichrome-staining
*OPG*: Osteoprotegerin
*OVX*: Ovariectomy
*P*: Phosphorus
*RANKL*: Receptor activator of NF-κB ligand
*RunX2*: Runt-related transcription factor 2
*TBT*: Trabecular bone thickness
*TRAP*: Tartrate-resistant acid phosphatase
